# No effect of natural transformation on the evolution of resistance to bacteriophages in the *Acinetobacter baylyi* model system

**DOI:** 10.1038/srep37144

**Published:** 2016-11-21

**Authors:** Amy McLeman, Pawel Sierocinski, Elze Hesse, Angus Buckling, Gabriel Perron, Nils Hülter, Pål Jarle Johnsen, Michiel Vos

**Affiliations:** 1 European Centre for Environment and Human Health, University of Exeter Medical School, Penryn Campus, Cornwall TR10 9FE, UK; 2 Department of Biosciences, University of Exeter, Penryn Campus, Cornwall TR10 9FE, UK; 3 Department of Biology, Bard College, Annandale-On-Hudson, NY 12574, USA; 4 Department of Pharmacy, UiT The Arctic University of Norway, 9037 Tromsø, Norway; 5 Institute of General Microbiology, Christian-Albrechts-University Kiel, Am Botanischen Garten 11, D-24118 Kiel, Germany

## Abstract

The adaptive benefits of natural transformation, the active uptake of free DNA molecules from the environment followed by incorporation of this DNA into the genome, may be the improved response to selection resulting from increased genetic variation. Drawing analogies with sexual reproduction, transformation may be particularly beneficial when selection rapidly fluctuates during coevolution with virulent parasites (‘the Red Queen Hypothesis’). Here we test this hypothesis by experimentally evolving the naturally transformable and recombinogenic species *Acinetobacter baylyi* with a cocktail of lytic phages. No increased levels of resistance to phage were found in the wild type compared to a recombination deficient Δ*dprA* strain after five days of evolution. When exposed to *A. baylyi* DNA and phage, naturally transformable cells show greater levels of phage resistance. However, increased resistance arose regardless of whether they were exposed to DNA from phage-sensitive or –resistant *A. baylyi*, suggesting resistance was not the result of transformation, but was related to other benefits of competence. Subsequent evolution in the absence of phages did not show that recombination could alleviate the cost of resistance. Within this study system we found no support for transformation-mediated recombination being an advantage to bacteria exposed to parasitic phages.

It has been recognized for a long time that bacteria are not purely clonal, but that they are also able to laterally transfer genetic information through uptake and recombination of foreign DNA^1^,^2^. One main mechanism of lateral gene transfer is natural transformation: the uptake of free DNA from the environment followed by its recombination into the genome during a physiological state termed competence^3^. Homologous recombination can create novel combinations of alleles and potentially speed up natural selection by alleviating clonal interference, analogous to meiotic sex in eukaryotes^4^,^5^.

Consistent with this hypothesis, natural transformation-mediated genetic exchange improved adaptation to a novel lab environment in *Helicobacter pylori*^6^, and enabled highly increased rates of antibiotic resistance evolution in experimental environments amended with single and multiple antibiotics in *Acinetobacter baylyi*^7^. Other studies emphasize the context-dependence of transformation benefits. For example, natural transformation was beneficial when *Streptococcus pneumonia* populations were exposed to periodic stress but not during benign experimental conditions^8^ and natural transformation proficient populations of *A. baylyi* adapted better to log-phase growth than transformation deficient strains but this benefit was offset by reduced performance during late stationary phase^9^. Other non-mutually exclusive hypotheses have also been presented and experimentally tested for the evolutionary benefits of competence. One hypothesis suggests that DNA is taken up to repair double stranded DNA breaks^10^,^11^,^12^, another states that DNA is taken up in order to contribute to cellular metabolism^13^, and a third suggest that competence is maintained by episodic selection for growth arrested competent cells and the occasional uptake of beneficial DNA from the environment^14^.

Only continual, strong selection pressures are expected to favor transformation-mediated recombination, as in well-adapted populations it is expected to decrease fitness by disrupting favorable gene combinations^15^. A prominent candidate hypothesis for strong and fluctuating selection pressure is that of parasite attack, where a new adaptation of one species (infectivity) selects for a counter adaptation in the other species (resistance), and so on and so forth^15^,^16^,^17^. A recent study demonstrating that experimental nematode populations able to outcross were better able to evolve resistance against bacterial parasites than were clonal control populations provides support this hypothesis^18^. As with all organisms, bacteria are heavily parasitized, notably by viruses (‘bacteriophages’ or ‘phages’). Lytic phages bind to a receptor on the bacterial cell surface, inject their genetic material into the cell and take over the bacterial cellular machinery to make multiple copies of themselves which are released through lysis of the bacterium. Co-evolutionary arms races observed between bacteria and phages (e.g. refs 19, 20, 21) thus could provide a potential selective pressure that could maintain natural transformation in bacterial populations. Indeed, there is evidence that phage resistance can be acquired through transformation of O1-antigens in *Vibrio cholera*^22^. Here we use recombinogenic and non-recombinogenic *A. baylyi* in the presence of lytic bacteriophages to test whether transformation-mediated recombination can result in increased levels of phage resistance, and/or whether it allows more efficient compensation of costly resistance mutations in the absence of phages.

## Results

### The effect of transformation on *A. baylyi* on resistance evolution to lytic phage

**Figure 1:**
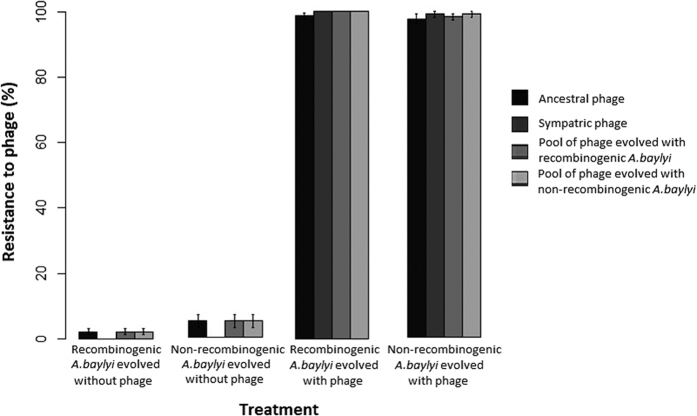
Resistance to phage after five days of evolution of recombinogenic *A. baylyi* BD413 wild type or non-recombinogenic Δ*drpA* mutant in the presence of a phage cocktail. Resistance against four phage pools (ancestral phage, evolved sympatric phage, phage evolved with all recombinogenic replicate lines and phage evolved with all non-recombinogenic replicate lines, see main text) represented as average percentage clones infected (out of 24 clones for six replicates). (Sympatric phage susceptibility could not be tested for the two control treatments that were not evolved with phages). Bars represent standard error bars.

In order to test whether the ability to recombine via transformation affects resistance evolution, wild-type and non-recombinogenic mutant clones of *A. baylyi* were evolved in the absence or presence of lytic phage. In contrast to our expectations, the recombinogenic wild type did not show higher resistance to phage than the mutant (Fig. 1). Although selection regimes differed significantly in terms of host resistance against ancestral phage, this pattern was driven by phage presence/absence rather than transformation ability (Kruskal-Wallis test: χ^2^_3_ = 18.48, *p*-value < 0.001) (Fig. 1). Post-hoc comparisons indicate no significant differences in resistance to phage between recombinogenic and non-recombinogenic evolved clones (Tukey test, *p*-value > 0.05). The same pattern was found for sympatric phages (evolved with bacteria in the same flask) (Kruskal-Wallis test: χ^2^_1_ = 2.2, *p*-value < 0.5) and for phages pooled from all six replicates evolving with either type of bacterium (Kruskal-Wallis test: χ^2^_3_ = 19.45 (phage pooled from recombinogenic lines), χ^2^_3_ = 19.51 (phage pooled from non-recombinogenic lines), *p*-value < 0.001). This result was not due to loss of transformability over the duration of the evolution experiment (see Supplemental Material).

### Short-term effects of recombination

**Figure 2:**
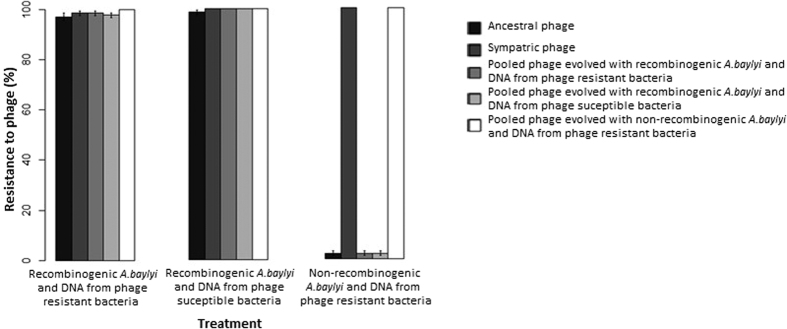
Resistance to phage within the wild type recombinogenic *A. baylyi* and the non-recombinogenic Δ*drpA* strain after overnight incubation in the presence of bacteriophage and DNA isolated from a mixture of six phage-resistant evolved clones, or control DNA isolated from the phage susceptible ancestor. For each of six replicates of each treatment, 24 clones were assayed for resistance against five phage populations (see main text). Bars represent standard error bars.

We next tested whether transformation provided an advantage to bacteria in the early phases of co-evolution. The recombinogenic and non-recombinogenic strains were incubated overnight in presence of the phage cocktail as well as 5 mg/ml DNA isolated from six evolved resistant clones. As a control, the recombinogenic strain was also incubated with the phage cocktail and 5 mg/ml of its own DNA. After overnight incubation, 24 clones from each replicate for each of the three treatments were assayed for resistance against five phage populations (ancestral phage, sympatric phage (from the same overnight microcosm), and pooled phage isolated from each of the three treatments) (Fig. 2). Recombinogenic cells gained significantly higher resistance to ancestral phage than non-recombinogenic cells after 24 hours (Kruskal-Wallis test: df = 2, χ^2^ = 12.511, *p*-value < 0.01), however the type of DNA provided had no significant effect on resistance (Tukey test, *p*-value > 0.05; Fig. 2). We found no significant difference for resistance to sympatric phage and phage isolated from pooled non-recombinogenic lines between treatments (Kruskall Wallis test, *p* value > 0.05).

### The effect of natural transformation on compensation for phage resistance

Although there is no indication in our system that homologous recombination mediated by transformation speeds up the spread of phage resistance alleles, it is possible that recombination could reduce clonal interference between mutations compensating for costly phage resistance mechanisms. In an overnight growth experiment, we could show that phage resistant clones of both strain types had significantly slower growth rate (V_max_) compared to phage sensitive clones (ANOVA, F_3,23_ = 48.20, *p*-value < 0.001). However, non-recombinant resistant clones had higher growth rate than recombinant resistant clones (ANOVA, F_3,23_ = 4.00, *p*-value < 0.05). To test whether in the absence of phage, recombinogenic clones could more readily compensate for costly phage resistance compared to non-recombinogenic clones, a subset of clones (eight clones from each of six replicates for each treatment) was evolved with a mixture of DNA from phage susceptible and phage resistant clones for ten overnight transfers. No support was found for recombination enabling a greater degree of compensation for costly phage resistance as growth rate at the final transfer was not found to be be significantly different between the two groups (χ^2^ = 0.36, d.f. = 1, *p*-value = 0.55).

## Discussion

Competence for natural transformation is increasingly characterized mechanistically in a variety of model bacterial species^23^ but the selective forces responsible for the evolution and maintenance of the ability to acquire exogenous DNA remain elusive^1^,^3^. The results presented here provide no evidence for the hypothesis that transformation-mediated recombination can speed up natural selection to gain resistance to lytic phages. This could be because the fast evolution of phage resistance observed here pre-empted any potential benefit of recombination. The co-evolutionary potential of bacteria has been experimentally shown to be greater than that of their antagonistic phage in different model systems^24^,^25^, and the observation that co-occuring phages can be largely non-infective on their hosts in nature is also consistent with this^26^. Differences between genetic bases of host resistance evolution in different bacterial types thus could result in different likelihoods of Red Queen-type dynamics. Alternatively or additionally, the experimental conditions used (e.g. bottleneck size or the presence of stress^9^) could have not been conducive to selection for transformation-derived adaptations. A previous study investigating the potential evolutionary benefits of recombination in *A. baylyi* found that the ability to transform was repeatedly lost over the course of experimental evolution^27^, however this was not observed here.

Phage resistance came at a higher cost for recombinant clones compared to non-recombinant clones. This could be due to a negative epistatic interaction between costs of recombination and resistance. Transformation-mediated recombination was not found to aid natural selection to overcome the cost of resistance after phages were removed. This could be due to the fact that such compensatory mutations readily occur, precluding any advantage of the exchange of alleles, as hypothesized above for the rapid evolution of phage resistance.

It is of course possible that competence and transformation in *A. baylyi* is not the result of selection to generate variation to increase the rate of adaptation. The variation in conditions that promote competence and the variation in how competence interacts with other cellular processes in different species points at the possibility that this process could have different and context-dependent roles in different species^3^. A variety of benefits have been shown to be associated with competence development^3^, including increased survival during short-term stress and increased genome stability during longer-term evolution under periodic stress^8^ in *Streptococcus*. The finding that transformation in *A. baylyi* resulted in increased short-term phage resistance regardless of the availability of DNA carrying resistance mutations could be consistent with benefits other than those conferred by recombination. A better understanding of resistance mechanisms and relevant ecological conditions in this system^28^, as well as the exploration of phylogenetically diverse model systems will be crucial to come to a better understanding of the adaptive roles of transformation.

## Methods

### Bacteria

Naturally competent *Acinetobacter baylyi* strain BD413 was used as the recombinogenic Wild Type. Strain BD413 carries the *trpE27* mutation, a G- > A transition in the *trpE* of the tryptophan biosynthesis pathway gene, causing auxotrophy for tryptophan^29^,^30^. To obtain a non-recombinogenic version of this strain, the DprA-encoding gene ACIAD0209 was inactivated by insertional inactivation. The DprA (DNA processing protein A) protein is a specific mediator for loading of the recombinase RecA onto internalized single-stranded DNA^31^ and is strictly required for chromosomal incorporation of exogenous DNA^32^. The *dprA* knock-out strain was constructed by replacing an internal 349-bp fragment of *dprA* with an *aacC1* encoding cassette (gentamicin resistance) using Splicing by Overlap Extension PCR (SOE-PCR)^33^. Briefly, chromosomal segments upstream and downstream of the desired insertion site in *dprA* were PCR-amplified from the chromosome of *A. baylyi* strain BD413 using primers dprA_1 and dprA_2 for the upstream segment (837-bp) and primers dprA_5 and dprA_6 for the downstream segment (851-bp) (Supplemental Table 1). Both fragments overlapped either end of a third PCR fragment containing the *aacC1* gene including its promoter (646-bp, amplified with primer aacC1_3 and aacC1_4 using plasmid pUC18T-miniTn7T-Gm-eyfp as template). The three primary PCR products were joined in a secondary-stage PCR reaction to generate a linear fragment containing the *aacC1*-marked gene insertion-deletion mutation (Δ*dprA*::*aacC1*). The resulting PCR product was directly used to naturally transform *A. baylyi* BD413. Transformants were scored on LB plates supplemented with gentamicin (4 μg ml^−1^). The Δ*dprA*::*aacC1* insertion-deletion mutation was confirmed by PCR for one of the transformants and the resulting strain was termed NH24. Inactivation of *dprA* in *A. baylyi* resulted in natural transformation frequencies below 1 × 10^−9^ transformants per recipient (unpublished data N. Hülter, V. Sørum, P. J. Johnsen).

### Phages

Phages were isolated by mixing a pool of environmental samples (soil, plant material, raw sewage) with 500 ml LB medium and 1 ml of an overnight *A. baylyi* BD413 culture. This enrichment culture was incubated overnight at 28 °C and shaken at 70 rpm. Enrichment culture samples (1 ml) were chloroformed and plated on soft agar overlays to check for plaques (see below). Single phage plaques were picked, re-amplified and picked again twice to ensure phage clonality and high phage density. Phage clones were stored at 4 °C. A phage cocktail consisting of four highly infective phage types diluted in equal measures to a final density of 10^8^ Plaque Forming Units (PFU)/ml was made for use in experiments. Phage types were selected based on their ability to infect the wildtype ancestor host after short-term evolution (Supplemental Material). This cocktail was also stored at 4 °C.

### Phage infectivity assay

Resistance to phage was assayed by growing individual bacterial clones overnight at 28 °C and mixing 300 μl of individual bacterial cultures with 7 ml soft (0.6%) LB agar followed by inverting the mixture ten times and allowing to set on hard (1.2%) LB agar. 5 μl of each phage treatment was spotted and allowed to dry on the soft agar overlay (ancestral phage was added as a positive control). Plates were incubated at 28 °C overnight and presence/absence of phage plaques were scored the next day.

### Evolution of phage resistance experiments

In a first experiment, both the recombinogenic and non-recombinogenic strains were transferred every 24 hours for five days in the presence of our phage cocktail. As a control, both strain types were also evolved in the absence of phage. The four treatments were replicated six times, yielding 24 experimental populations in total. Plastic microcosms with six ml 10% Luria Broth (LB) were inoculated with ~10^6^ Colony Forming Units (CFU) and ~10^6^ phage cocktail PFU in two treatments (Multiplicity of Infection (MOI) ~1:1 cells). The experiment was performed in diluted (10%) LB broth in order to maximize the cost of phage resistance^34^. Microcosms were kept in an incubator at 28 °C and shaken continuously at 180 rpm, with 1% culture volume transferred to a fresh microcosm every day. After approximately 33 bacterial generations (five transfers), whole phage populations were chloroformed (250 μl chloroform to 2.5 ml culture, gentle vortexing and spinning down for 5 min at 14.000 rpm) followed by filter sterilization of the supernatant (0.45 μm filter) and stored at 4 °C. Bacterial clones (*n* = 24 per replicate) were isolated by randomly picking colonies from dilution plates, growing up in 500 μl LB broth, adding glycerol to 20% final concentration and storing at −80 °C. Using a phage infectivity assay (see below), isolated clones were tested for resistance against: a) the ancestral phage cocktail, b) the phage isolated from the same microcosm (sympatric), c) phage pooled from all six replicates evolved with the recombinogenic line and d) phage pooled from all six replicates evolved with the non-recombinogenic line.

High levels of bacterial phage resistance evolved within five days in both strains (see Results). We determined whether recombination could provide an advantage over a shorter time scale, aided by the presence of DNA encoding phage resistance. The recombinogenic and non-recombinogenic strains (10^6^ CFU total) were incubated overnight in 2.5 ml 10% LB in presence of the phage cocktail (10^6^ PFU total) and 5 mg/ml DNA isolated from six evolved resistant clones (three wild type clones evolved with phage and three (rare) resistant wildtype clones evolved in the absence of phage). As a control, the recombinogenic strain was also incubated with the phage cocktail and 5 mg/ml DNA isolated from itself (all treatments n = 6). After overnight incubation, 24 clones from each replicate for each of the three treatments were assayed for resistance against five phage populations (ancestral phage, sympatric phage (from the same overnight microcosm), and pooled phage isolated from each of the three treatments).

### Transformation frequency assay

A phenol:chloroform:isoamyl alcohol DNA isolation protocol modified from Sambrook and Russel^35^ was used to obtain genomic DNA from the non-recombinogenic mutant containing the gentamicin marker. A Nanodrop 2000 (Thermo Scientific) and Qubit 2.0 (Invitrogen, Life Sciences) were used to verify DNA quality and quantity respectively (260/280 nm ratio ~1.8, 260/230 nm ratio ~2.0). Transformation frequency of the recombinogenic strain was assayed by inoculating 5 ml 10% LB with 50 μl of an overnight culture and adding 500 ng/ml of genomic DNA of the Δ*dprA::aacC1* mutant containing a gentamicin marker. After overnight incubation at 28 °C and 180 rpm, cells were plated on plain LB agar as well as LB agar supplemented with 5 μg/ml gentamicin (Amresco); transformation frequency was calculated by dividing CFU count of the latter by CFU count on the former.

### Cost of resistance evolution experiment

To measure the cost of phage resistance, all clones isolated at the end of the five-day evolution experiment were grown in 200 μl LB broth in a 96 well plate at 28 °C for 24 hours (in the absence of phage). The optical density was measured at 600 nm every hour to quantify growth rate (Varioskan Flash plate reader, Thermo Scientific). In a second experiment, designed to test whether recombination can speed up the evolution of compensation of costly phage resistance, a subset of recombinogenic and non-recombinogenic clones that were either phage resistant or phage sensitive were evolved in a 96 well plate containing 132.5 μl 10% LB, 1mg/ml DNA and a total of 10^6^ CFU of overnight culture at 28 °C for ten daily 5% transfers (in the absence of phage). The DNA added was isolated from a mixture of evolved (phage resistant) clones and the ancestral (phage susceptible) recombinogenic clone (Supplemental Material). The optical density was measured at 600 nm every hour to quantify final density.

### Data analysis

Tests were performed in the package R version 3.0.1^36^, unless stated otherwise. The initial five-day experiment testing the effect of recombination on evolving phage resistance was analyzed using a non-parametric Kruskal Wallis test for each phage cocktail separately, with 4 levels (recombinogenic with and without phage and non-recombinogenic with and without phage). In case of significant main effects, we used Tukey post hoc comparisons, with α < 0.05. To test whether clones evolved in this experiment, recombinogenic or non-recombinogenic and either phage resistant or phage sensitive (n = 24 per treatment), differed in their cost of resistance, we used a one-way ANOVA in JMP 11 (Statistical Discovery™)^37^. Using a Wilcox test, we tested for the effect of DNA source (three levels) on phage resistance evolution in the non-recombinogenic and recombinogenic strains during overnight incubation. To test for the effect of recombination on compensatory costs we used a linear mixed effects model (LME; lmer function in lme4 package)^38^ with strain as fixed effect and random intercepts fitted for each replicate. The significance of the explanatory variable was established using likelihood ratio tests, which were χ^2^ distributed.

## Additional Information

**How to cite this article**: McLeman, A. *et al*. No effect of natural transformation on the evolution of resistance to bacteriophages in the *Acinetobacter baylyi* model system. *Sci. Rep.* **6**, 37144; doi: 10.1038/srep37144 (2016).

**Publisher’s note**: Springer Nature remains neutral with regard to jurisdictional claims in published maps and institutional affiliations.

## Supplementary Material

Supplementary Information
