# Supplementary material for: No effect of natural transformation on the evolution of resistance to bacteriophages in the Acinetobacter baylyi model system
Source: Sci Rep. 2016 Nov 21;6:37144. doi: 10.1038/srep37144 (PMC5116665; doi:10.1038/srep37144)
Supplement: Supplementary Information [file srep37144-s1.pdf]

Supplemental Material "No effect of natural transformation on the evolution of resistance to bacteriophages in the *Acinetobacter baylyi* model system"

Amy McLeman, Pawel Sierocinski, Elze Hesse, Angus Buckling, Gabriel Perron, Nils Hülter, Pål Jarle Johnsen and Michiel Vos.

Primers

Supplemental Table S1

Name	5' – 3'
dprA_1	TCAATCTGTCCCACATCACCAG
dprA_2	GCCCTAGGCTCCCATACCACGCGCGCCTTGAAACAGCACATACGAG
dprA_5	CCGCTAGCAATTACCTACCGGCGTTCTTTTCGAATTGCTGCTGTCC
dprA_6	AGGTGTATGTTCGTGGTCAAGG
aacC1_3	GCGCGTGGTATGGGAGCCTAGGGCTGTTAGGTGGCGGTACTTGG
aacC1_4	ACGCCGGTAGGTAATTGCTAGCGGGGC**TTGAC**AGCAGCAAGCGCGTTA**TAAT**GTGGGTCG

Complementary 5' overhangs of primers used for SOE-PCR (as described in the text) are underlined. Primer aacC1_4 introduced nucleotide exchanges (added nucleotides shown in bold caps) in the 5' region 59 to 35 nucleotides upstream of *aacC1*, thereby generating a new promoter derived from the CP6 promoter (Jensen and Hammer 1998; Poteete et al. 2006). The new promoter replaced the gene's distantly located native promoter from Tn*1996* (Wohlleben et al. 1989).

Lytic *A.baylyi* phage cocktail

Phages could be isolated from six different enrichment cultures. One phage clone from each enrichment culture was amplified on the *A. baylyi* wild type strain for three overnight transfers and re-isolated. All six evolved phage lines were spotted on 24 clones from each of the six evolved bacterial cultures to determine (cross-)infectivity (Table S2). Infectivity patterns were used as an indicator of phage type. Phages from populations A, B, C and D have distinct infectivity patterns on evolved bacterial clones, indicating that they are genetically different. Phage A shows high infectivity while the bacteria it evolved with did not gain effective resistance. Phage B shows high infectivity while the bacteria it evolved with gained poor but variable resistance. Phage C shows high infectivity while the bacteria it evolved with gained 40% resistance to phage. Phage D shows high infectivity but the bacteria it evolved with evolved complete resistance to all phage. Phage E and F are no longer infective to evolved clones. For our phage cocktail (ϕC), equal volumes of phages A, B, C and D were mixed to produce a cocktail at a concentration of 10^8 PFU.

Supplemental Table S2. Percentage of bacterial (cross-)resistance to phages isolated from six different enrichment cultures (A-F) each evolved on the same ancestral bacterial clone.

		Phage					
		**A**	**B**	**C**	**D**	**E**	**F**
**Bacteria**	**A**	100	100	100	100	0	0
	**B**	93	93	93	88	0	0
	**C**	60	60	60	60	0	0
	**D**	0	0	0	0	0	0
	**E**	82	77	82	77	0	0
	**F**	95	95	95	95	0	0

Cocktail of bacterial DNA for the cost of resistance experiment

A mixture of DNA from evolved (phage resistant) clones and the ancestral (phage susceptible) clone was provided to both recombinogenic and non-recombinogenic phage resistant cells evolving in the absence of phage. Half of the mix consisted of DNA isolated from the recombinogenic ancestor. Half of the mix consisted of DNA originating from a mixture of all resistant clones from our five-day evolution experiment; this included clones from our treatments evolved with phage (144 clones from the recombinogenic strain and 141 clones from our non-recombinogenic strain) as well as several clones evolved without phage (three evolved from the recombinogenic strain and seven clones evolved from the non-recombinogenic strain). All individual clones were grown separately overnight in 96-well plates and mixed in equal volumes prior to DNA isolation.

Testing possible loss of transformability in the five-day evolution experiment

Six evolved wild type clones (six replicates of three clones evolved with phage and six replicates of three clones evolved without phage) were compared to six replicates of the ancestral recombinogenic clone. A one-way ANOVA test was carried out in R version 3.0.1[35] based on the average of the replicates for each type ($F_{2,9}$ =0.21, p-value=0.82).
